# To compare the effectiveness of constraint induced movement therapy versus motor relearning programme to improve motor function of hemiplegic upper extremity after stroke

**DOI:** 10.12669/pjms.315.7910

**Published:** 2015

**Authors:** Sana Batool, Nabila Soomro, Fareeha Amjad, Rabia Fauz

**Affiliations:** 1 Sana Batool, MSPT. Institute of Physical Medicine & Rehabilitation, Dow University of Health Sciences, Karachi, Pakistan; 2 Dr. Nabila Soomro, FCPS. Institute of Physical Medicine & Rehabilitation, Dow University of Health Sciences, Karachi, Pakistan; 3 Fareeha Amjad, MSPT. Institute of Physical Therapy, University of Lahore, Lahore, Pakistan; 4 Rabia Fauz, MSc. PT. Institute of Physical Medicine & Rehabilitation, Dow University of Health Sciences, Karachi, Pakistan

**Keywords:** Constraint movement therapy, Motor learning programme, Physiotherapy, Rehabilitation, Stroke

## Abstract

**Objective:**

To compare the effectiveness of constraint induced movement therapy versus motor relearning programme to improve motor function of hemiplegic upper extremity after stroke.

**Method:**

A sample of 42 patients was recruited from the Physiotherapy Department of IPM&R and Neurology OPD of Civil Hospital Karachi through non probability purposive sampling technique. Twenty one patients were placed to each experimental and control groups. Experimental group was treated with Constraint Induced Movement Therapy (CIMT) and control group was treated with motor relearning programme (MRP) for three consecutive weeks. Pre and post treatment measurements were determined by upper arm section of Motor Assessment Scale (MAS) and Self Care item of Functional Independence Measure (FIM) Scale.

**Results:**

Intra group analysis showed statistically significant results (p-value<0.05) in all items of MAS in both groups. However, advanced hand activities item of MAS in MRP group showed insignificant result (p-value=0.059). Self-care items of FIM Scale also showed significant result (p-value< 0.05) in both groups except dressing upper body item (p-value=0.059) in CIMT group and grooming and dressing upper body items (p-value=0.059 & 0.063) in MRP group showed insignificant p-values.

**Conclusion:**

CIMT group showed more significant improvement in motor function and self-care performance of hemiplegic upper extremity as compared to MRP group in patients with sub-acute stroke assessed by the MAS and FIM scales. Thus CIMT is proved to be more statistically significant and clinically effective intervention in comparison to motor relearning programme among the patients aged between 35-60 years. Further studies are needed to evaluate CIMT effects in acute and chronic post stroke population.

## INTRODUCTION

Stroke is the third leading cause of death and the most common cause of disability in the developed and developing countries. In Pakistan it is the leading cause of mortality and morbidity after cancer and ischemic heart disease.^1^ The number of expected cases with stroke in our country is 250/100,000 each year, progressing to 350,000 new cases annually. Burden of stroke is escalating in Asia and incidence ranges from 182 to 342 per 10,000 populations.^2^ Upper extremity motor impairment is the most disabling consequence of stroke that limits independent living.^3^ About 85% of stroke population exhibits an initial weakness in arm that persists in 55% to 75% of patients even after three to six months.^4^ However, full recovery of hemiplegic arm occurs in only 5 to 20% of patients.^5^ Hemiplegic arm reduces the ability to actively perform functional arm movements such as reaching, grasping and manipulating an object that result in difficulties in activities of daily livings (ADLs).^3^

Rehabilitation of upper extremity is a challenge, various therapeutic strategies like neurodevelopmental technique (NDT), splinting, biofeedback and electrical stimulation are designed, however the best method to improve upper limb function is still not clear.^6^ Two widely practiced therapies to improve motor function of hemiplegic upper extremity are motor relearning programme and constraint induced movement therapy (CIMT). Motor relearning programme focuses on task specific learning through effective use of feedback and practice and studies have shown that it is effective in enhancing motor function recovery of post stroke paretic limb.^7^,^8^ However, CIMT is a more recent intervention and has gained much attention in the treatment of post stroke paretic arm.^3^ After stroke, patients are incapable to use the hemiplegic arm thus compensate by using the unaffected arm more, leading to learned non use phenomenon. CIMT helps to overcome learned non use by constraining movement of unaffected arm with a sling or mitt and encourage repetitive task practice of affected arm including shaping, a behavioral technique.^4^ A number of studies reported that CIMT significantly improves functional activities and maximizes motor function of paretic upper limb.^3^,^4^,^9^

This study is a randomized controlled trial carried out to evaluate the effectiveness of CIMT over motor relearning programme. Constraint induced movement therapy (CIMT) is proved to be effective in western countries^4^ but the evidence for this kind of intervention is lacking in Pakistan. Thus the aim of this study was to investigate the effectiveness of CIMT over motor relearning programme to improve motor function of hemiplegic upper extremity after stroke.

## METHODS

In this randomized controlled trail 42 males and females stroke patients having first attack of stroke and hemiplegic upper extremity had functional level of ≥ 20 degrees of wrist extension and ≥10 degrees of extension of all digits were included. Subjects were between 35-60 years of age and diagnosed with ischemic or hemorrhagic strokes confirmed by CT/MRI scan. Patients were excluded if they had cognitive deficit, recurrent or cerebellar strokes, severe aphasia, balance and equilibrium problems, severe shoulder pain affecting therapy or other neurological deficits.

The research was started after approval from institutional review board (IRB) of Dow University of Health Sciences (DUHS). Sample was recruited from Physiotherapy department of Institute of Physical Medicine & Rehabilitation (IPM&R) and Neurology OPD of Civil Hospital Karachi through non-probability purposive sampling technique. Informed consent was taken from the patients. Then patients were randomly allocated to CIMT group and MRP group by using computer-generated random numbers kept in sealed envelopes. Each group had 21 patients. After baseline assessment, patients in CIMT group were asked to perform tasks only with the hemiplegic upper extremity with the unaffected hand restraint in a mitt. The patients in MRP group were asked to perform tasks with both affected and unaffected upper extremities. Tasks were attempted in different positions like supine lying, sitting and standing.

Both interventions consisted of 6 sessions per week, two hours duration of each session, for three consecutive weeks. Pre and post treatment evaluation of motor function of hemiplegic upper extremity was determined by using upper arm section of Motor Assessment Scale. Upper limb section of MAS includes, Upper arm function item, hand movements item and advanced hand activities item. An activity of daily livings (ADLs) was measured by using self care item of Functional Independence Measure Scale (FIM) which includes 5 items (eating, grooming, bathing, dressing upper body and dressing lower body. Outcome of both treatment groups was re-evaluated on 18^th^ visit. All the exercises were demonstrated to the patients by the therapist and were performed under the supervision of the therapist. Details of exercises are given in Table-I.

**Table-I:** Exercises demonstrated to CIMT & MRP Groups.

Experimental Group (Constraint Induced Movement Therapy)	Control Group (Motor Relearning Programme)
The participants in this group were allowed to perform tasks only with the hemiplegic upper extremity with the unaffected hand restraint in a mitt.	The participants in this group were allowed to use their both upper extremities (affected & unaffected) for exercises.
The participants in this group underwent repetitive task practice exercises like stacking cones and stacking blocks, grasping and releasing of an object, reaching forward and sideways, lifting and dropping of an object from the one end of table to the other end. Task complexity was gradually increased using behavioral techniques of shaping.	The participants in this group underwent different task oriented exercises like reaching and pointing activities, weight bearing of hemiplegic upper extremity & practice of different bimanual tasks in sitting positions, like holding the jar with unaffected hand and opening the lid with affected hand and vice versa.

### Statistical Analysis

SPSS version 16 was used for data analysis. Statistical results were expressed as mean ± standard deviation (S.D) for quantitative data. A Non parametric Wilcoxon Signed Ranks test was conducted to find the significance of interventions used within the groups (Intra-group analysis). To test the significance of changes in scores on each of the outcome measures (MAS scale, FIM scale) between the groups (Inter-group analysis), a Non parametric Mann Whitney test was used both at the baseline and at the end of 3^rd^ week. The significance level of all comparisons was set at p-value < 0.05.

## RESULTS

A sample of 42 patients participated in the study and no patient dropped out. There were total 28 male participants (66.67%) and 14 (33.33%) female participants in both groups, the distribution of males and females in each groups was almost equal. The mean age of the participants was 49.67 years (SD = 7.01) in CIMT group and 49.47 years (SD = 8.19) in MRP group. Total patients who suffered from ischemic stroke were 32 (76.2%) and number of patients having hemorrhagic stroke were 10 (23.8%) respectively. At the end of third week, mean scores significantly increased in all items of both outcome measures (MAS and FIM) in both CIMT and MRP groups. However, mean value in CIMT group was greater than in MRP group, showing better improvement in CIMT group (Table II & III). Intra-group analysis showed statistically significant results (p-value<0.05) in all items of MAS in both groups. However, advanced hand activities item of MAS in MRP group showed insignificant result (p-value=0.059). All self-care items of FIM Scale also showed significant result (p-value< 0.05) in both groups, however dressing upper body item (p-value=0.059) in CIMT group and grooming and dressing upper body items (p-value=0.059 & 0.063) in MRP group showed insignificant result (Table II & III).

**Table-II:** Pre and Post treatment mean and standard deviation for all items of MAS & FIM scale in CIMT group.

Outcome Measures	Pre-treatment (Mean±S.D)	Post-treatment (Mean±S.D)	P-value
MAS: Upper arm function	1.33±0.48	5.00±0.63	0.001*
MAS: Hand Movements	0.81±0.40	4.05±0.74	0.001*
MAS: Advanced hand activities	0.28±0.46	1.57±1.07	0.002*
MAS: Total (Max Score:18)	2.43±.81	10.62±1.20	0.001*
FIM: Eating	1.34±0.48	5.62±0.58	<0.001*
FIM: Grooming	1.19±0.40	4.05±0.49	<0.001*
FIM: Bathing	1.24±0.44	3.90±.62	<0.001*
FIM: Dressing upper body	1.38±0.49	1.71±0.78	0.059
FIM: Dressing Lower body	1.28±0.46	3.57±0.51	<0.001*
FIM: Total (Max score: 35)	6.43±1.56	18.86±1.52	<0.001*

* Statistically significant; MAS=Motor assessment scale; FIM=Functional independence measure.

**Table-III:** Pre and Post treatment mean and standard deviation for all items of MAS & FIM scale in MRP group.

+0Outcome Measures	Pre-treatment (Mean±S.D)	Post-treatment (Mean±S.D)	P-value
MAS: Upper arm function	1.33±0.48	4.19±0.68	<0.001*
MAS: Hand Movements	0.76±0.44	2.33±1.32	0.002*
MAS: Advanced hand activities	0.38±0.50	0.62±0.50	0.059
MAS: Total (Max Score:18)	2.57±1.16	7.14±1.56	<0.001*
FIM: Eating	1.34±0.48	4.62±0.50	<0.001*
FIM: Grooming	1.24±0.44	1.48±0.75	0.059
FIM: Bathing	1.24±0.44	3.48±0.51	0.001*
FIM: Dressing upper body	1.38±0.50	1.62±.067	0.063
FIM: Dressing Lower body	1.34±0.48	3.00±0.55	0.001*
FIM: Total (Max score: 35)	6.52±1.72	14.19±1.47	0.001*

* Statistically significant; MAS=Motor assessment scale; FIM=Functional independence measure.

For inter-group analysis a non parametric Mann Whitney test was used at baseline and at the end of 3^rd^ week. Post treatment P-value showed statistically significant results (p-value < 0.05) in all items of MAS and FIM Scale accept dressing upper body item.

## DISCUSSION

This study showed the effectiveness of constraint induced movement therapy (CIMT) over motor relearning programme (MRP) to improve motor function of hemiplegic upper extremity in sub-acute stroke patients. The affected patients in the present study were between two weeks to three months post stroke. CIMT initiated within 2 weeks of stroke onset is more effective in upper extremity rehabilitation.^10^ Present study results further support the view that functional recovery after stroke possibly can occurs after three months to at least six months.^11^,^12^

This study results are in agreement with Myint et al. (2008)^9^ study which suggested significant improvements were found in motor function of hemiplegic arm after 2 weeks of intervention and 12 weeks follow up in CIMT group. However, in present study no follow up was carried out after 3 weeks. Taub et al. (2006)^13^ study findings also showed considerable improvements in CIMT group as compared to placebo group. In this study mean age of study participants was 54.6 years in CIMT group while in control group it was 50.7 years. However, in the present study the mean age of the participants was 49.67 and 49.47 years in the CIMT and MRP groups respectively, almost close to above study. Results of the present study showed improvements in both groups but significantly in CIMT group. Difference in the results might be due to the reason that present study included younger age group patients. However, in other CIMT studies older stroke population was selected with the mean age 66.4 years in one study^14^, while in another study mean age was 63.4 years in CIMT group and 63.9 years in control group.^9^

In the present study an alteration was made from the original protocol^15^ by reducing the treatment time from 6 to 2 hours per day to permit for local service structure. The author has changed the intensity protocols by distributing total treatment time over a longer duration. Wu et al. (2007)^16^ also used the same protocol as used in the present study and showed good results. The author proposed that MRP given to control group may require a longer time period to show maximal improvements. This statement is supported by Chan et al. (2006)^8^ study which suggested that six weeks of MRP revealed better improvement in terms of functional performance on self-care, instrumental activities of daily living and integration into the community.

CIMT is considered to attain its beneficial effects by 2 associated but independent mechanisms: overcoming learned nonuse and use-dependent neural plasticity.^17^ Converging evidence showed that learned non use is a learning phenomenon which causes inhibition of the movement that initiates in the acute period after the central nervous system damage. This Learned inhibition of movement is overcome by a behavioral technique called shaping used in CIMT.^18^

The second mechanism by which CIMT has been established to give good functional results is use-dependent neural plasticity.^17^ It is associated with the large alterations in function that CIMT generates in humans after stroke and monkeys after simulated stroke.^19^ The motor relearning approach focuses on task specific learning through effective use of therapist’s feedback to enhance the recovery of motor skills of hemiplegic arm in post stroke patients.^7^,^8^ Conversely CIMT is based on the theory of learned non use and forcing the use of hemiplegic arm in post stroke hemiparesis by immobilizing the normal arm in a sling or mitt and encourage repetitive task practice of hemiplegic arm including shaping, a behavioral technique used in CIMT.^20^

## CONCLUSION

CIMT group showed more significant improvement in motor function and self-care performance of hemiplegic upper extremity as compared to MRP group in patients with sub-acute stroke assessed by the MAS and FIM scales. Thus CIMT is proved to be more statistically significant and clinically effective intervention in comparison to motor relearning programme among the patients aged between 35-60 years. Further studies are needed to evaluate CIMT effects in acute and chronic post stroke population.
